# Prediction and Construction of Energetic Materials Based on Machine Learning Methods

**DOI:** 10.3390/molecules28010322

**Published:** 2022-12-31

**Authors:** Xiaowei Zang, Xiang Zhou, Haitao Bian, Weiping Jin, Xuhai Pan, Juncheng Jiang, M. Yu. Koroleva, Ruiqi Shen

**Affiliations:** 1 College of Safety Science and Engineering, Nanjing Tech University, Nanjing 211816, China; 2 School of Chemistry and Chemical Engineering, Nanjing University of Science and Technology, Nanjing 210094, China; 3 Jiangxi Xinyu Guoke Technology Co., Ltd., Xinyu 338018, China; 4 Institute of Modern Energetics and Nanomaterials, D. Mendeleev University of Chemical Technology of Russia, Moscow 125047, Russia; 5 Micro-Nano Energetic Devices Key Laboratory of MIIT, Nanjing 210094, China; 6 Institute of Space Propulsion, Nanjing University of Science and Technology, Nanjing 210094, China

**Keywords:** energetic material, machine learning, materials discovery and prediction, data augmentation, computer-learned representation

## Abstract

Energetic materials (EMs) are the core materials of weapons and equipment. Achieving precise molecular design and efficient green synthesis of EMs has long been one of the primary concerns of researchers around the world. Traditionally, advanced materials were discovered through a trial-and-error processes, which required long research and development (R&D) cycles and high costs. In recent years, the machine learning (ML) method has matured into a tool that compliments and aids experimental studies for predicting and designing advanced EMs. This paper reviews the critical process of ML methods to discover and predict EMs, including data preparation, feature extraction, model construction, and model performance evaluation. The main ideas and basic steps of applying ML methods are analyzed and outlined. The state-of-the-art research about ML applications in property prediction and inverse material design of EMs is further summarized. Finally, the existing challenges and the strategies for coping with challenges in the further applications of the ML methods are proposed.

## 1. Introduction

Developing and exploring advanced EMs with high energy, low sensitivity, and good thermostability today remains a challenge [1,2,3,4,5,6,7,8,9,10]. In general, the high energy of EMs is always accompanied by increased mechanical sensitivity and decreased thermostability [1,3,8]. EMs research has historically relied heavily on either trial-and-error processes or serendipity, which require a great deal of tedious experimentation [2,5,11,12]. Many of these intuition-based approaches are inefficient and time-consuming, and they can be costly and risky [2,4,12,13]. Currently, the classical paradigm of material R&D is still based on the method of “putting forward hypothesis—experimental verification”, to continuously approach the target material [14,15,16].

In addition to the experiments, computational chemistry has also become a mature approach to complement and aid experimental studies for predicting and designing novel EMs [2,12,16,17,18,19,20,21,22,23,24], such as the density functional theory (DFT) method [25,26]. Several empirical models have been developed to guide the EMs design, including the Kamlet-Jacobs equation and the nitro charge method [27,28]. However, to accurately calculate the microstructure parameters and properties of materials, computational chemistry methods require a large number of calculations by high-performance computers [1,2,29,30]. Even though the computing power of modern computers has been huge, in the face of multi-scale calculation of complex properties of materials, computational chemistry methods require substantial computing resources, and the time and economic costs are very high [1,2,30,31].

The ML method is extracting patterns and insight from data and finding the statistical law behind the data to produce reliable, repeatable decisions and results [13,16,21,26,32,33,34,35,36,37,38,39]. Physical insight and mechanisms were used extensively to construct classical models, such as conservation laws and thermodynamics for regressing linear or slightly nonlinear parameters [16,40]. The ML method takes a different route: instead of relying on principles or physical insights, it relies on data and algorithms [26,41]. As big data are becoming more readily available, data-driven or ML methods have opened new paradigms for the discovery and rational design of materials [41]. By applying ML methods, R&D costs for advanced materials can be reduced, and the R&D speed of advanced materials can be increased [24,42,43,44,45,46,47,48,49,50,51]. The application of ML methods in the research field of EMs has gradually received more and more attention [2,24,52,53]. For example, Nguyen et al. [24] aimed to predict the crystalline density of a class of EMs known as high explosives (HE) by the ML method.

A large number of systematic reviews have been written on the application of the ML method in material research, such as in lithium-ion batteries [54], mechanical metamaterials [55], catalysts [56,57,58], nanoparticles [59], and in field of pyrolysis, thermal analysis, and thermokinetic studies [60]. On the contrary, a relatively small number of reviews have been published on applying the ML method in the research field of EMs [61]. Herein, this review mainly focuses on the scientific progress of ML applications in EMs over the last decade. First, a brief workflow on various ML methods is put forward, and we describe the main ideas and basic procedures for employing ML approaches. We then highlight the state-of-the-art research about the applications of ML for property prediction and the novel EMs discovery. In the last section, we discuss various challenges regarding the development of ML methods for EMs, and ideas for addressing them. Lastly, conclusions are presented along with an outlook.

## 2. ML Workflow

**Figure 1:**
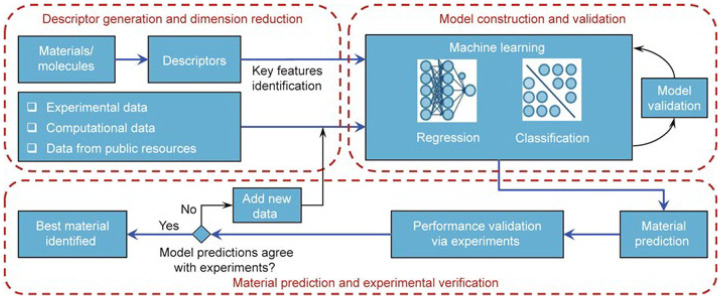
Illustration of ML workflow [16]. Copyright 2019 Elsevier.

Generally speaking, the workflow of ML is to build models based on reliable data and suitable features, to optimize the models continuously, and to predict and design the target eventually, as illustrated in Figure 1.

As shown in Figure 1, the basic steps for applying ML methods include data preparation, feature engineering, model construction, and model performance evaluation [16,62]. However, the application steps of ML methods will vary according to the different research objects. Thus, in this review, we describe the main ideas and basic procedures for employing ML approaches for EMs property prediction and inverse material design.

### 2.1. Data Preparation

It is common for ML-based applications in EMs to begin with the construction of new datasets and/or the utilization of existing datasets. It is recommended that the datasets are divided into three parts, namely, the training datasets for training the model, the verification datasets for parameter adjustments, and the test datasets for testing the model.

Data is the key to effective ML application. The data in the dataset mainly consists of experimental results, computational results, and data from the literature. Song et al. [1] gathered more than 1000 pieces of EMs data from the literatures to train the property of regression models. A wide variety of molecules were included in the dataset, including aliphatics, aromatics, monocyclics, and polycyclics [1]. To accelerate the discovery of energetic melt-castable materials, Song et al. [63] collected more than 1000 pieces of data from the literature to construct a structure-property dataset for ML model training. Chandrasekaran et al. [64] compiled a dataset, which consists of 104 data points for a wide range of carbon, hydrogen, nitrogen, and oxygen (CHNO) explosives at different loading densities, using experimental data available in the literature [64].

Nguyen et al. [24] curated a dataset of energetic-like molecules from the Cambridge Structural Database (CSD) and sub-selected from the database molecules that either are known HE or are similar to this family of compounds by imposing several restrictions [24]. To train the classification model, Song et al. [1] prepared 365 entries indicating not graphite-like and 22 entries indicating graphite-like from the Cambridge Crystallographic Data Centre (CCDC). Casey et al. [65] procured molecules from the GDB database [66,67] to consider only those with “energetic potential” according to oxygen balance (OB). Walters et al. [68] used the void size distribution to quantify key features of the microstructure and the hydrodynamic reaction rate across a range of shock pressures to measure the initiation performance of EMs. Then they used the reactive flow model working in the hydrodynamic solver system to generate the training dataset [68]. The common databases in the pieces of literature are shown in Table 1.

A sufficient quantity, quality, and diversity of data were necessary for ML methods, and results could be impressive when sufficiently large datasets are available [2]. However, for data preparation in the research field of EMs, setting up an extensive database is impractical as the available datasets are limited and difficult to collect. In particular, the amount of data was too small and unsuitable for the deep learning method. However, generative ML models must be able to handle small datasets to solve project-tailored design tasks in EMs research. In such cases, data augmentation has been proposed as an effective strategy to work in small-data regimes and obtain reliable results for the research of EMs and other materials [34,77,78].

Moret et al. [34] augmented the data using the simplified molecular input line entry specification (SMILES) enumeration trick, which generated multiple different SMILES strings that represented the same molecule. To reliably screen the potential EMs with a high detonation velocity, Li et al. [79] also utilized the SMILE enumeration augmentation to build a recurrent neural network (RNN)-based prediction model. SMILES enumeration, as proposed by Arús-Pous et al. [80], is an important data-augmentation technology for molecular deep learning. In addition, given the problem of data scarcity, Elton et al. [2] would like to challenge the assumption that large datasets are necessary for the ML method to be useful by doing the comparison of ML methods to energetic data. Elton et al. [2] focused on a small but diverse dataset consisting of 109 molecular structures spread across 10 compound classes. The scholars did this using a dataset of 109 energetic compounds computed by Huang and Massa [2,29]. While they later introduce additional data from Mathieu [81] for most of their work, they restrict their study to the Huang and Massa data to demonstrate how well different ML models and featurization work with small data.

Due to the diversity of data sources for ML models, data fidelity is important in constructing reliable and accurate ML models [82,83]. For example, ML models developed using low-fidelity data will be limited in accuracy [82,83]. Thus, in addition to the frequently-used data augmentation approach mentioned above, there is also a noticeable method developed to overcome data scarcity in materials science. Patra et al. [84] introduced the multi-fidelity (MF) information fusion approach to build powerful prediction models of polymer bandgaps. The MF information scheme that utilizes information available at different levels of fidelity could be a more optimal way to build predictive surrogate models [84]. In principle, the MF information fusion approach could also be used in the data preparation of ML for the prediction and construction of novel EMs. 

In the applications of ML methods for EMs, the low data regime is typical data development environments. Data augmentation, reasonable feature selection, and model construction are the critical strategies for successfully applying ML methods in a small data environment.

### 2.2. Feature Engineering

An effective ML model requires developing suitable machine-readable representations [36,65]. The machine-readable representations were commonly called “descriptors”, “features”, “fingerprints”, or “profiles” [36,65]. It was possible to improve the predictive power of ML models without having an extensive database by selecting features based on the physicochemical nature of the target properties [73].

**Figure 2:**
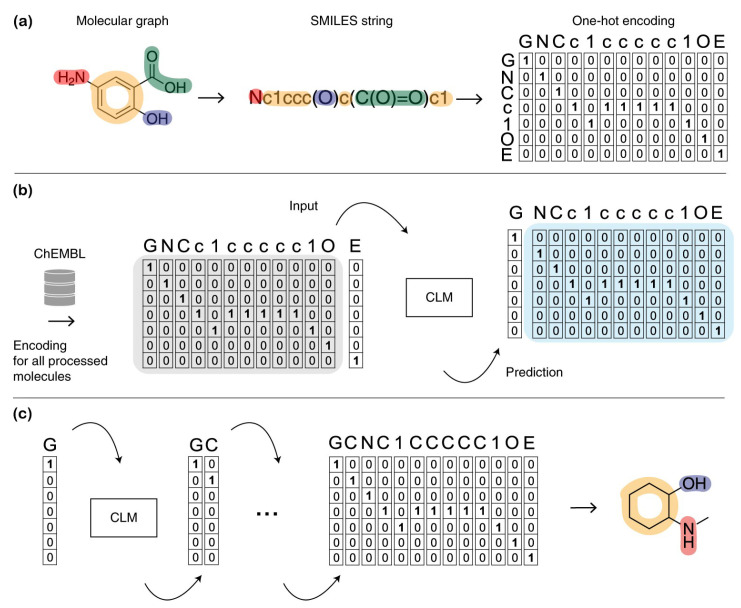
Chemical language model training and sampling of new molecules. (**a**) Each molecule is translated into a SMILES string. (**b**) The chemical language model learns the feature distribution of the dataset. (**c**) The chemical language model repeatedly samples tokens from the learned distribution [34]. Copyright 2020 Springer Nature Limited.

In the research field of materials science, how to quantitatively represent molecules is the key to implementing the ML method [36,85,86,87]. Since the 1970s, molecular representations have evolved from chemical informatics models [88]. Chemical databases were scanned for structural similarity using fast bitwise logic using fingerprints, which encode molecular 2D substructures as overlapping lists of patterns [88]. For example, a common approach to representing molecules with fixed-length bit vectors corresponds to the presence or absence of features using E3FP [88] and ECFP [89]. Song et al. [1] constructed the halogen elements from the electron-topological state fingerprint [90,91,92], which has been widely used to construct different models for predicting molecular properties. The SMILES representation was also developed to encode the structure of a chemical species into short ASCII strings, making it suitable for text-based models [13,26,30,93,94], as shown in Figure 2.

Decades of research have gone into developing effective descriptors to index a large number of molecular structures [95]. For example, Xie et al. [13] considered four types of descriptors to characterize the molecular structure, such as sum over bonds, extended connectivity fingerprint, E-state fingerprint, and custom descriptor set. This is especially relevant as numerous investigations have shown that the molecular descriptor selection can influence model accuracy more than the choice of the ML algorithm [1,2,24,56,65].

#### 2.2.1. Traditional Class of Molecular Representation

**Figure 3:**
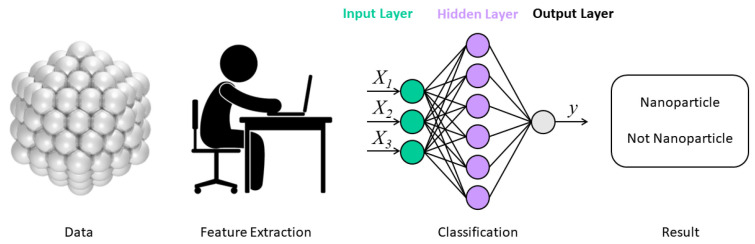
To predict the size distribution into a special structural identification of nanoparticle-like material by the classification model, the method of simple neural networks is used where researchers manually undertook feature extraction [96]. Copyright 2019 The Royal Society of Chemistry.

In general, a descriptor is a set of features that are manually derived and incorporate domain knowledge about chemical properties to provide necessary information about molecular structures [95]. For example, RDKit is an open-source toolkit for chemical informatics [13,92]. This approach was suitable for traditional ML approaches that require a predetermined set of engineered features [24]. The traditional feature extraction undertaken by researchers is illustrated in Figure 3.

Custom descriptors were defined to enhance descriptions of molecular shapes, energetic characteristics, and interactions between molecules [63]. Song et al. [1] defined a custom descriptor set containing 29 molecular descriptors, which are related to the elements of carbon, hydrogen, oxygen, and nitrogen [1]. With this custom descriptor set, researchers will be able to describe molecular shape and composition, such as the plane of best fit and OB, so that they can learn more about EMs’ properties [1]. Wang et al. [4] constructed the descriptors of a molecule including elementary percentage, OB, substituent kind and number, and type of two adjacent substituents [4].

A comprehensive comparison of several molecular featurization methods, including the sum over bonds, custom descriptors, coulomb matrices, bag of bonds, and fingerprints was presented by Elton et al. [2]. The first descriptor they chose was OB [2]. Next, the nitrogen/carbon ratio was chosen [2], which is a well-known predictor of energetic performance [97]. Substituting nitrogens for carbon generally increases performance since N=N bonds yield a larger heat of formation/enthalpy change during detonation compared to C-N and C=N bonds [97]. Moreover, Elton et al. [2] stated that with small data, significant gains in accuracy can sometimes be achieved by hand-selecting features using chemical intuition and domain expertise. For example, the number of azide groups in a molecule was known to increase energetic performance while also making the compound more sensitive to shock [2].

To efficiently extract the desired physicochemical properties from a relatively small database, Chen et al. [73] proposed the concept of spatial matrix descriptors. Under this concept, volume occupation spatial matrix and heat contribution spatial matrix were constructed as descriptors for ML models to feature spatial distribution of mass and energy of the energetic molecules in atomic view to predict the crystalline density and solid phase heat of formation [73]. The idea behind spatial matrices was to reduce redundant information concerning target properties in the coulomb matrix by adding proper physical-chemical causality relationships.

The bulk modulus (mechanical property) and the impact sensitivity are crucial for energetic compounds. However, relationships have not been elucidated between the molecular structure, the bulk modulus, and the two important properties. Deng et al. [74] obtained 17 molecular descriptors for impact sensitivity as the target property, including eight classes composed of 2D autocorrelations, geometrical descriptors, descriptors, atom-centered fragments, etc. It was found that the main contributions of the descriptors to the impact sensitivity come from the geometric distance between oxygen atoms, the number of oxygen-containing double bonds, hydrophilicity and the distribution of atomic properties [74].

#### 2.2.2. Computer-Learned Representation

**Figure 4:**
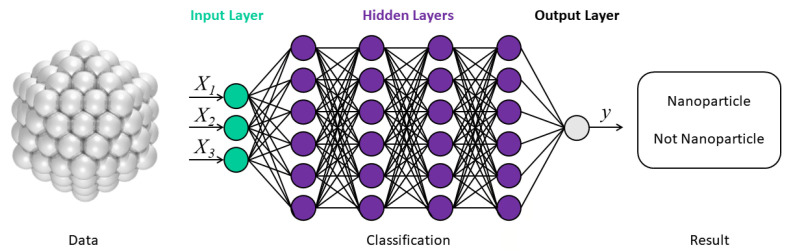
To predict the size distribution into a special structural identification of nanoparticle-like material by the classification model, the method of deep neural networks is used where feature extraction is automatically undertaken in additional hidden layers by artificial intelligence [96]. Copyright 2019 The Royal Society of Chemistry.

Generative deep learning methods represent a class of ML algorithms that learn directly from the input data and do not necessarily depend on explicit rules coded by humans [34]. For example, deep learning networks are capable of learning rich data representations [34,65], which provided a compelling motivation to use deep learning networks to learn molecular structure-property relations from “raw” data [65]. The computer-learned representation is illustrated in Figure 4.

**Figure 5:**
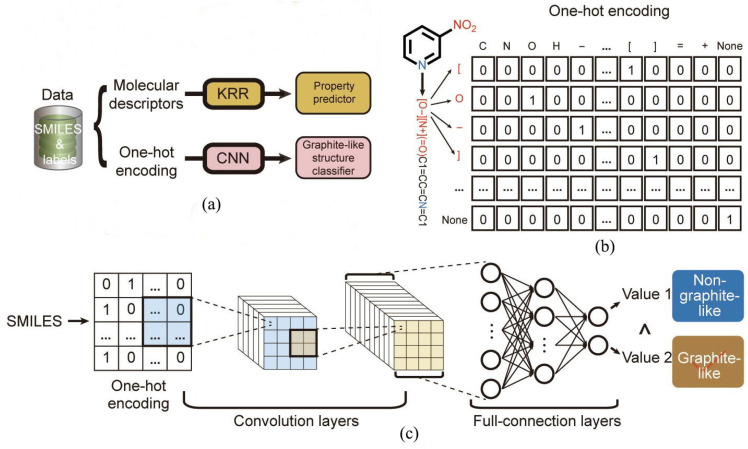
Framework and components of the system. (**a**) Schematic of the training of property models (kernel ridge regression-KRR) and the graphite-like structure classification model. (**b**) One-hot encoding for the input of the CNN. (**c**) Architecture of the CNN [1]. Copyright 2022 Elsevier LTD.

Song et al. [1] developed a more reliable method for screening potential energetic compounds with low sensitivity. Since there is a widely recognized close correlation between graphite-like layered crystal structure and low-impact sensitivity in EMs [9,69,98], Song et al. [1] tried to translate the direct prediction of impact sensitivity into a special structural identification of graphite-like layered crystal packing. Accordingly, the convolutional neural network (CNN) and long short-term memory (LSTM) [99,100] were chosen to capture the chemical intuition necessary to distinguish among molecules regarding possible graphite-like crystal structures. The framework is shown in Figure 5.

As seen in Figure 5, the CNN was trained using the one-hot encoding of the SMILES strings [93,94] as input [1]. A comparison of the training process indicates that the SMILES_Onehot + CNN model was better than the other models. Beyond selecting molecules of interest, CNN requires that each molecule has an associated “input” and “output”. To bypass feature selection, a CNN was proposed to learn a mapping directly from the molecule electronic structure and is described as a 3D spatial point data for charge density and electrostatic potential stacked into a 4D tensor [65]. This method effectively bypasses the need to construct complex representations, or descriptors, of a molecule. To capture the main driving force to crystallization, Jiang et al. [72] developed a graph neural network (GNN) model-based deep learning framework to predict the formation of the co-crystal. This model outperformed seven competitive models and three challenging independent test sets involving pharmaceutical co-crystals, π–π co-crystals, and energetic co-crystals with greater than 96% accuracy [72].

In the application process of the ML method, molecular representations are the bridge and link between data and the model algorithm. With the development of deep learning methods in recent years, computer-learned representation has more advantages than traditional class feature extraction [1,24,70,71,72,74,79,101]. The main disadvantage of deep learning is that the amount of computational power required depends heavily on the number of samples, and on the number of hidden layers and sophistication of the network [96]. For the specific physical quantities, the prediction errors of the computer-learned representation and traditional class feature extraction were summarized [1,24,70,71,72,74,79,101]. The prediction performance of the computer-learned representation and the traditional class feature extraction for certain physical quantities is summarized in Table 2 for better comparisons.

As shown in Table 2, to more reliably screen the molecules with a high detonation velocity, the SMILE enumeration augmentation coupled with the pretrained knowledge was utilized to build an SRNN prediction model, through which R^2^ was boosted from 0.9445 to 0.9572 [79].

### 2.3. ML Models in EMs Prediction and Construction

ML model and algorithm are inseparable. ML algorithms can be broadly classified into supervised and unsupervised learning algorithms. The supervised learning algorithm may be further classified into regression and classification. In material design, by using a set of known materials and their properties, a supervised learning algorithm attempts to identify a function that can predict the properties of novel materials. The process is known as regression if the target property is continuous. Classification is identifying the prediction function when the outputs are discrete targets. By using unsupervised learning methods, such as clustering, input data are identified as having a relationship among themselves. A list of important ML methods in the literature is shown in Table 3.

As seen in Table 3, ML methods adopted in the literature can be classified as the supervised learning algorithm. Moreover, some methods are in the category of traditional ML models, others are deep learning methods, and all the methods are in the category of regression and classification.

#### 2.3.1. The Regression Models

**Figure 6:**
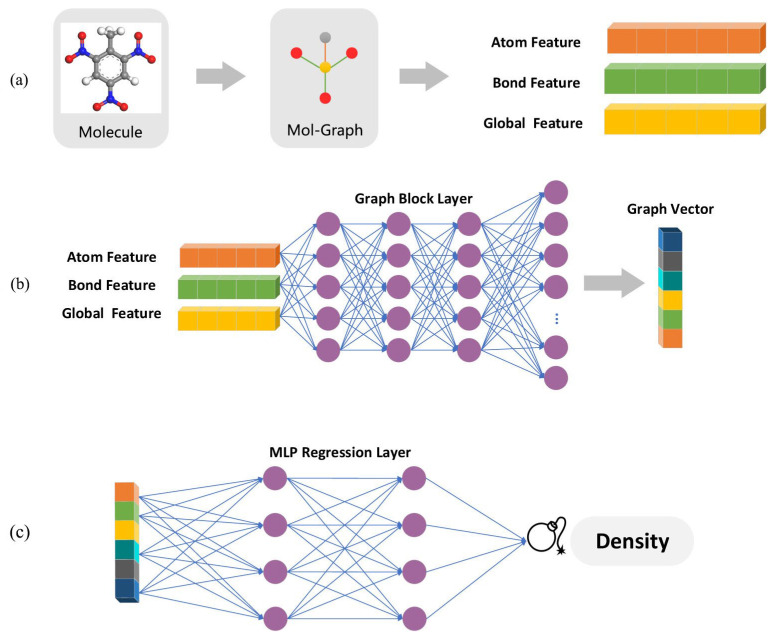
The framework of the density prediction model. (**a**) Extracting features from molecular topologies. (**b**) Vectorising features via a graph block layer. (**c**) Regressing via an ANN model [70]. Copyright 2021 American Chemical Society.

The density and enthalpy of formation are measures of how much energy is stored in the EMs [5,70]. Density is an important indicator because it is directly related to the detonation velocity [24]. Detonation velocity is one of the basic indicators of the performance of explosives and is related to the fundamental elemental and structural properties of the explosives [64]. To directly characterize energetic performance, the heat of the explosion was also used as the target property [76]. The prediction of such properties was of great interest to those dealing with the EMs synthesis [64]. For example, the reported heterocyclic EMs possess increased densities, high enthalpies of formation, and high stability to various forms of external stimuli [5]. The framework of the density prediction model [70] is shown in Figure 6.

As shown in Figure 6, the model training process was implemented by using a multilayer ANN model [70]. The conventional SVM and RF models were also employed to build QSPRs between the molecular topology and crystal density [70]. The GNN-based model has higher accuracy and lower computational resource cost than the widely accepted DFT−QSPR model [70]. Using a database containing 451 energetic molecules, Chen et al. [73] showed that volume occupation spatial matrix and heat contribution spatial matrix can improve the accuracy in predicting EMs’ crystal density and solid phase enthalpy. Their mean absolute errors were reduced from 0.048 g·cm^−3^ and 24.67 kcal·mol^−1^ to 0.035 g·cm^−3^ and 9.66 kcal·mol^−1^, respectively.

**Figure 7:**
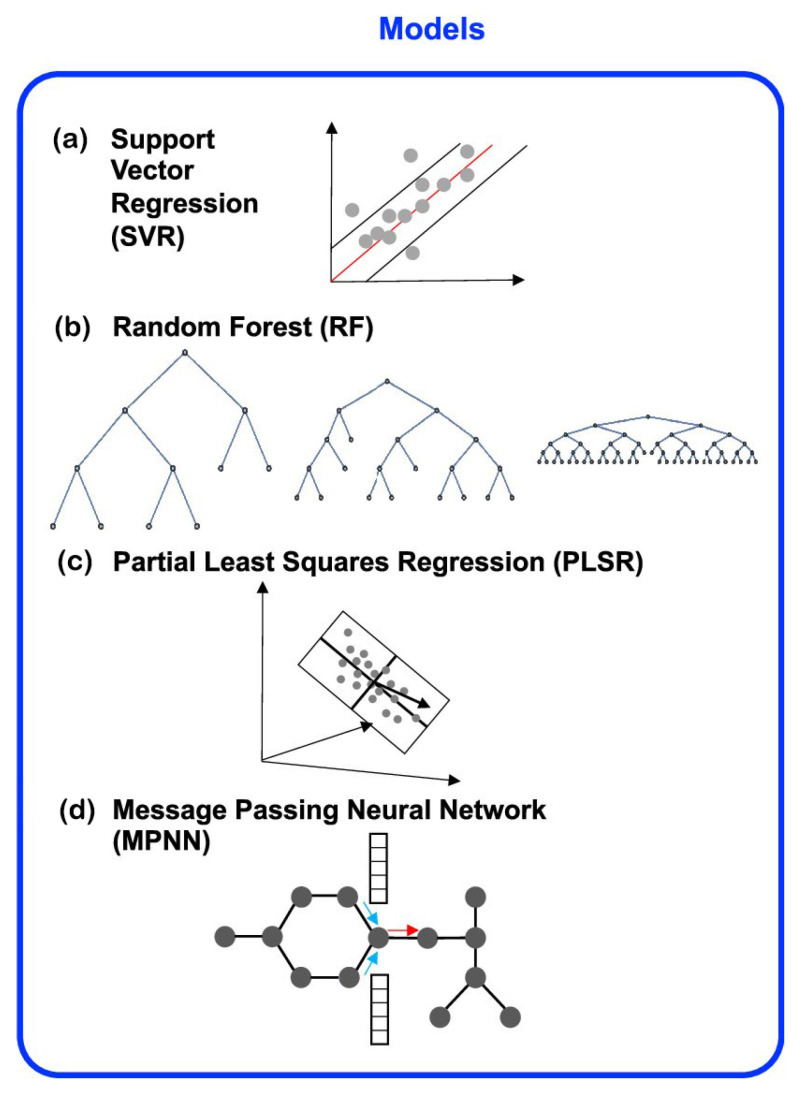
Overview of density regression models [24]. Copyright 2021 American Chemical Society.

Nguyen et al. [24] focused on several regression-based methods, which are compatible with the molecular-level featurization methods of RDKit and the E3FP fingerprints [24]. Nguyen et al. [24] developed and evaluated: (1) an MPNN-based model, which utilizes RDKit atom- and bond-level features to describe network nodes (atoms) and edges (bonds) but yields a learned overall molecular representation; (2) RF- and partial least-squares regression (PLSR)-based models with RDKit molecular-level features and (3) a SVR model using E3FP fingerprints. The results showed that the MPNN-based models with computer-learned molecular representations generally perform best, outperforming the RF and SVR models at predicting crystalline density and performing well even when testing on a dataset not representative of the training data. It was demonstrated that, despite the absence of crystal structure information or quantum mechanical calculations, the ML method can learn relationships between crystalline properties of molecules and chemical structures [24]. The overview of density regression models [24] is shown in Figure 7.

**Figure 8:**
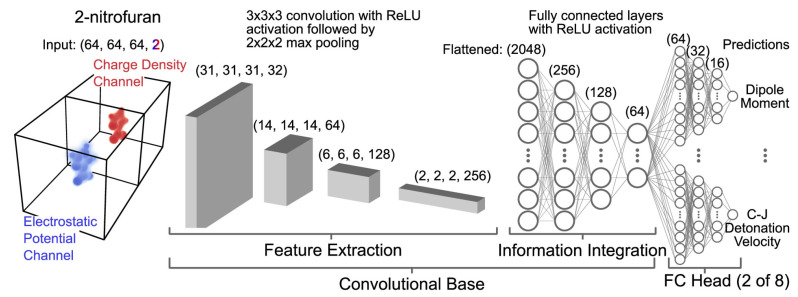
Selected model architecture. An example molecule, 2-nitrofuran, is represented by a standardized input [65]. Copyright 2020 American Chemical Society.

An approach to using the ANN technique to predict the detonation velocity had been attempted by Chen et al. [102]. However, Chen et al. [102] considered only the chemical composition of CHNO for predicting detonation velocity [102]. The CNN model has jointly trained on over 20,000 molecules that are potentially EMs to predict dipole moment, total electronic energy, Chapman−Jouguet (C−J) detonation velocity, C−J pressure, C−J temperature, crystal density, and solid phase heat of formation [65]. The selected model architecture [65] is shown in Figure 8.

As shown in Figure 8, this architecture shares a convolutional base that greatly reduce the number of inputs seen by the final eight fully connected layer blocks [65]. Additionally, joint learning provided a means for the network to learn a richer set of representations [65]. The 3D CNN model, without any parameter tuning, outperformed tuned RF models using extended-connectivity fingerprints. This model attained an excellent generalization error even when making predictions on structurally dissimilar molecules, as observed with scaffold-based splitting [65]. Chandrasekaran et al. [64] developed two ANN models. Model 1 showed that it can predict the detonation velocity of a wide range of CHNO explosives at various loading densities, the effect of density on detonation velocity, as well as possible predictions of detonation velocity in unexplored environments. In Model 2, the N and O composition of C, H, N, and O-based explosive molecules could be predicted for a targeted detonation velocity. Chandrasekaran et al. [64] presented the possible usage of the ANN method for predicting detonation velocity that can be of use in EMs research.

#### 2.3.2. The Classification Models

Compared with the regression model, the application of the classification model in ML methods for developing advanced EMs was relatively less but mainly focused on the prediction of the sensitivity of EMs. For decades, it has been known that high-performance explosives are characterized by high impact sensitivity, i.e., low values of the drop weight impact height H_50_ [81]. Zhang et al. [28] developed and established a method of calculating the Mulliken net charges of the nitro group, Q_NO_2__, to assess impact sensitivities for nitro compounds. The result [28] showed that the charges on the nitro group could be regarded as a structural parameter to estimate the impact sensitivity on the bond strength, OB, and molecular electrostatic potential. The nitro compound with more -Q_NO2_ will be insensitive and have a large impact sensitivity H_50_ value. This method considering the molecular structure was applicable for almost all nitro compounds when the C-NO_2_, N-NO_2_, or O-NO_2_ bond is the weakest in the molecule. According to the results, the nitro compounds with -Q_NO2_ > 0.23e show H_50_ ≤ 0.4 m [28].

In recent years, the ANN technique has been used for the prediction of impact sensitivity of EMs [74,103,104,105,106]. Materials with high energies and low-impact sensitivity usually have π−π stacking in conjunction with hydrogen bonding. A rather large π-bond is a requisite for the π−π stacking, and the π−π stacking can be classified into four patterns, including face-to-face stacking, wavelike stacking, crossing stacking, and mixing stacking [3]. The results [9] also indicated that the layer-by-layer geometries of high-performance insensitive EMs can readily absorb mechanical stimuli by converting kinetic energy into layer sliding, resulting in lower sensitivities. Deng et al. [74] found a significant correlation between the impact sensitivity and the bulk modulus, which is mainly dependent on the number of C, H, O, and N atoms, the molecular weight, and the OB by using the ANN and other models. Nowadays training a general model for sensitivity is still difficult, since sensitivity is correlated with multiscale factors, including the electronic structure, crystal structure, and even measurement conditions [1,69]. Therefore, an alternative method for tackling sensitivity prediction remains highly desired [1,69].

### 2.4. Model Performance Evaluation

An ML model can memorize data points in the training set, and thus result in extremely high accuracy regarding these data during the model testing. For this reason, ML models must be evaluated based on the new dataset that has not been used for training.

#### 2.4.1. Model Evaluation in the Regression Model

**Figure 9:**
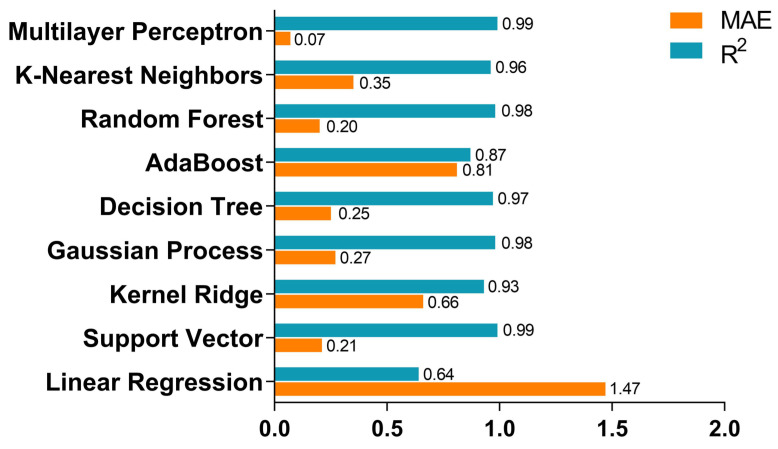
MAE losses and R^2^ scores of each regression method using a five-feature dataset with an 80% training set size [101]. Copyright 2022 American Chemical Society.

It is common to use the test dataset prepared in data preprocessing to test the model. Because the test dataset is completely new to the model, it can objectively measure the model’s performance in the real world. Specifically, a key point of the ML regression model is how to evaluate the accuracy of the model, which is described by “fitting degree”. Common evaluation indicators in regression learning include the mean absolute error (MAE), the root mean square error (RMSE), and the determination coefficient (R^2^) [1,2,4,13,24,53,63,65,68,70,75,76,79]. The scholars [1,16,24] applied stratified k-fold cross-validation to fairly assess the ML models. For example, for handling the density imbalance and ensuring that each fold represents the distribution of densities, Nguyen et al. [24] defined five stratified folds with bins between 1.0 and 2.0 at increments of 0.05. For each ML method adopted, the researchers summarized its overall performance by computing the averages of the R^2^ score and RMSE across the stratified folds [24]. As an alternative to stratified splitting, scaffold splitting may also be used to evaluate a method’s ability to generalize to structurally different molecules [24]. The MAE losses and R^2^ scores of the different regression methods is shown in Figure 9.

As seen in Figure 9, to achieve this benchmark, both MAE loss and R^2^ score were plotted by comparing the test losses for the nine selected supervised methods. The MLP and SVR methods gave the highest accuracy (MAE < 0.2 m·s^−1^) and the highest R^2^ scores (0.985 for the SVR method and 0.994 for the MLP method). The linear regression and AdaBoost algorithms offered the lowest accuracy (MAE ~1.4 m·s^−1^ and 0.87 m·s^−1^, respectively) and worst R^2^ score (0.636 and 0.875, respectively), which meant that compared to burn rate variance, the mean square error is too high [101].

#### 2.4.2. Model Evaluation in the Classification Model

To evaluate the classification performance of a model, it is necessary to introduce some evaluation indicators. The commonly used indicators include accuracy, precision, recall, F value, etc. [1,107]. In the classification model evaluation, the precision value measures the reliability of a model’s positive predictions, and the recall value measures its ability to find all the true positive sample points. The F value is the harmonic mean of the precision and recall values [107,108]. When there are more than two classes, there is a precision, recall, and F_1_ score for each class, characterizing a model’s ability to distinguish a specific class from all others. Taking the binary classification problem as an example, the scholars [1,107] largely used the F_1_ score as it provides a single score, largely independent of the choice of threshold, making the comparison between two models straightforward.

## 3. Applications of ML in R&D of EMs

### 3.1. Single-Compound EMs

Besides the property prediction discussed in this review, the vital purpose of ML methods in R&D of EMs is rational reverse material design. The goal of the inverse material design is to find promising advanced materials which were not known before and prior to lab experiments [109]. Kang et al. [76] identified 262 CHNO-based compounds with an 2,4,6-trinitrotoluene (TNT) equivalent power index *P*_e(TNT)_ greater than 1.5 as potential candidates for EMs, by combining the ML methodologies, materials informatics, and thermochemistry. To raise *P*_e(TNT)_ further to larger than 1.8, 29 potential candidates were found, and all are new to the current reservoir of well-known EMs. To directly characterize energetic performance, the heat of explosion was used as the target property [76]. A forward stepwise selection from a large number of possible descriptors led to critical descriptors for cohesive energy averaged over all constituent elements, plus OB [76]. Using the critical descriptors, even though the ML dataset is small, a satisfactory surrogate ML model was trained, with estimates R^2^ = 0.93 and MAE = 142.12 kJ·.kg^−1^ for the test dataset [76].

For a long time, nitrobenzene compounds were the focus of novel EMs research [4]. Two distinctive nitrobenzene compounds are hexanitrobenzene (HNB) and 1,3,5-triamino-2,4,6-trinitrobenzene (TATB). In terms of energy content, HNB and TATB are highly energetic. For example, the density of HNB is 1.988 g·cm^−3^, and the detonation velocity of TATB is 7825 m·s^−1^ [4]. In terms of insensitivity, TATB possesses a lower sensitivity to heat, and impact compared to HNB, and the bond dissociation energy of TATB is 304 kJ·mol^−1^ [4]. Wang et al. [4] decoded HNB and TATB by the ML method, in combination with theoretical calculations to predict the target properties, such as the density, the heat of formation, bond dissociation energy, and molecular flatness. The results showed that HNB was the most energetic compound among 370,000,000 single-benzene ring-containing compounds, while TATB displayed a moderate energy level and very high safety level and was also determined experimentally [4]. 

**Figure 10:**
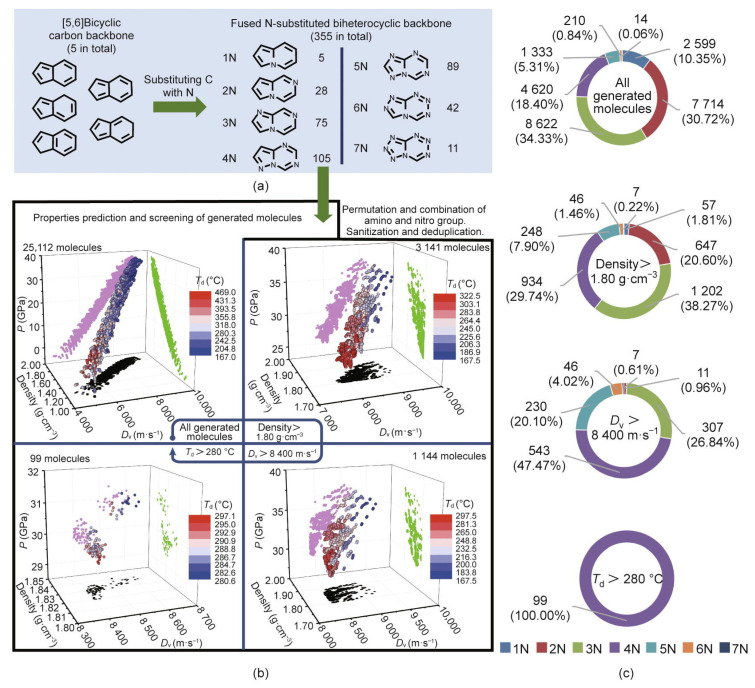
Process of generating and screening the molecules. (**a**) Illustration of the generation process. (**b**) Color-mapped 3D scatter plots of the molecules in original and different screening steps. (**c**) Proportions of other nitro-atom-substituted fused [5,6] biheterocyclic molecules in original and different screening steps [1]. Copyright 2022 Elsevier.

Fused heterocycle ring-based materials have also gained increasing attention in recent years [1], and researchers have reported the discovery of a series of promising fused-ring energetic molecules [1,6,7,10,12,110]. Herein, using a fused [5,6]biheterocyclic backbone and substituted nitro/amino groups, Song et al. [1] first constructed energetic molecules. Next, using a ML-assisted high-throughput virtual screening (HTVS) system, the discovery of novel EMs with well-balanced energy-safety properties was accelerated. In the HTVS system, Song et al. [1] used homemade scripts, and generated molecules through a heuristic enumeration method [26,111]. With the HTVS system, the promising target molecules from 25,112 generated molecular structures were rapidly filtered out. The promising targets also possess a relatively high likelihood of having graphite-like crystal structures. The process of generating and screening the molecules is shown in Figure 10.

**Figure 11:**
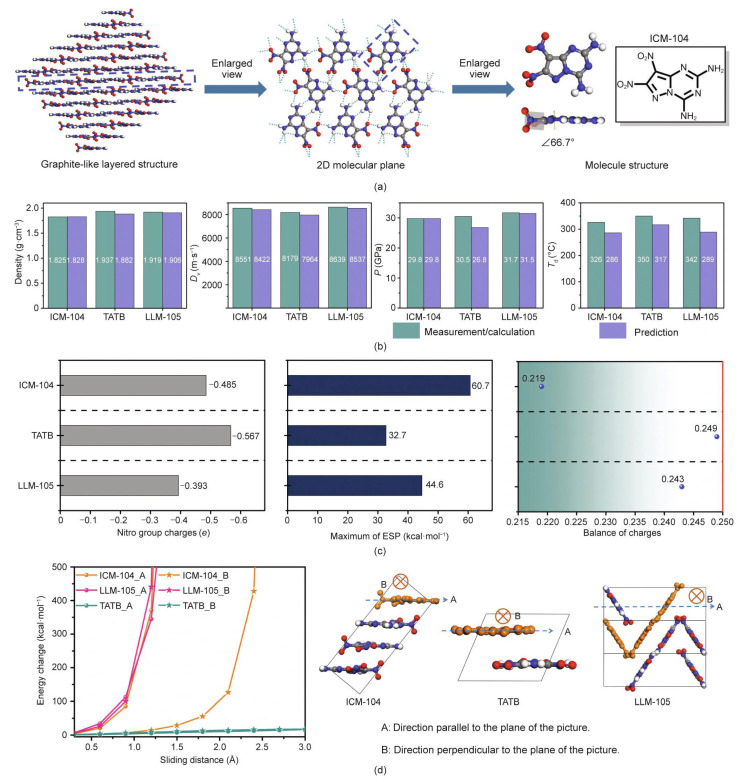
Crystal structure and properties of ICM-104. (**a**) Three-dimensional graphite-like layered crystal stacking, 2D supramolecular plane, and molecular geometry of ICM-104. (**b**) Comparison between the predicted and measured/calculated properties of ICM-104, TATB, and 2,6-diamino-3,5-dinitropyrazine-1-oxide (LLM-105). (**c**) Comparison of nitro group charges, maximum of electrostatic potential, and balance of charges of ICM-104, LLM-105, and TATB (1 kcal = 4.19 × 10^3^ J). (**d**) Energy change for the layer sliding of ICM-104, LLM-105, and TATB [1]. Copyright 2022 Elsevier.

As shown in Figure 10, the promising fused [5,6]bi-heterocyclic backbone-based compound-namely 7,8- dinitropyrazolo[1,5-a][1,3,5]triazine-2,4-diamine (ICM-104)-was successfully synthesized in the lab [1]. The crystal structure and properties of ICM-104 is shown in Figure 11.

**Figure 12:**
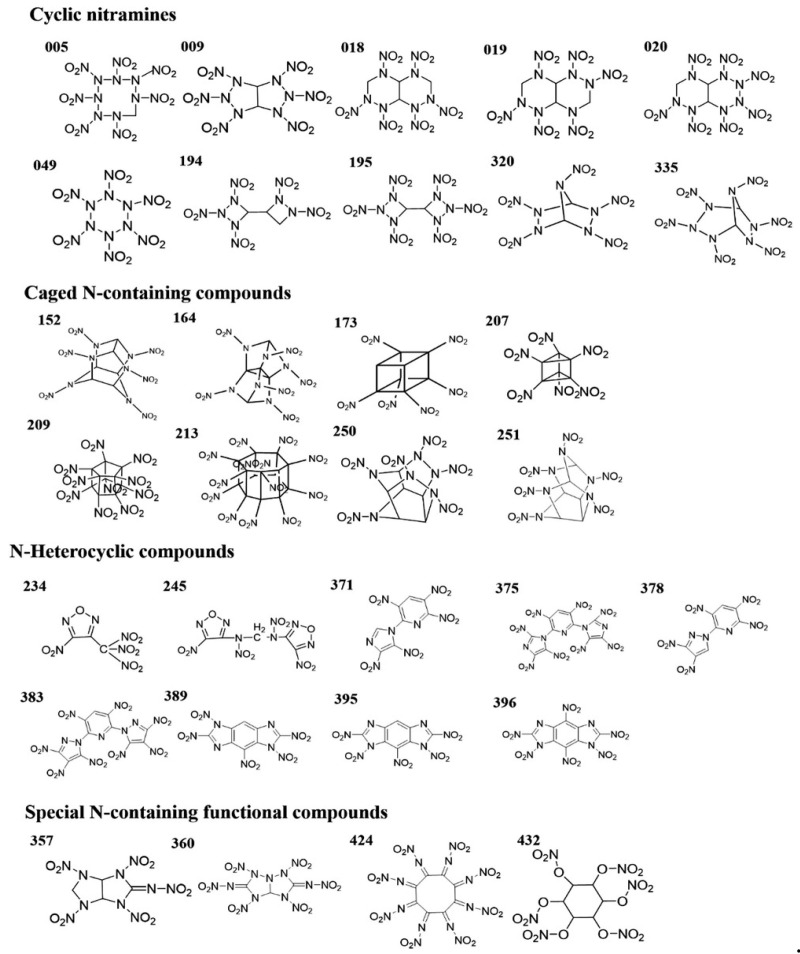
Molecular structures of the as-screened 31 N-containing molecules [23]. Copyright 2021 Wiley-VCH GmbH.

The novel compound has high energy properties, a low sensitivity, and good thermostability according to a study of its properties [1]. Using fused-ring energetic molecules as their research object, Wang et al. [53] obtained skeletons with high density through skeleton pre-screening, and then through fragment docking created a virtual screening space with molecules with high density. Quantum chemical calculations and equations of the state of detonation products were used to predict enthalpy of formation, detonation performance, and chemical stability. Finally, based on performance ranking, six novel energetic molecules with energy levels superior to 1,3,5-trinitro-1,3,5-triazinane (RDX) and stability superior to TNT were selected [53]. Hou et al. [23] established the neural network model to achieve the prediction and screening tasks. The screening criteria for potential advanced EMs was set to be density ≥ 1.9 g·cm^−3^, detonation velocity ≥ 9000 m·s^−1^, and detonation pressure ≥ 40.0 GPa. After screening, 31 novel N-containing molecules with outstanding detonation properties were found, as shown in Figure 12.

**Figure 13:**
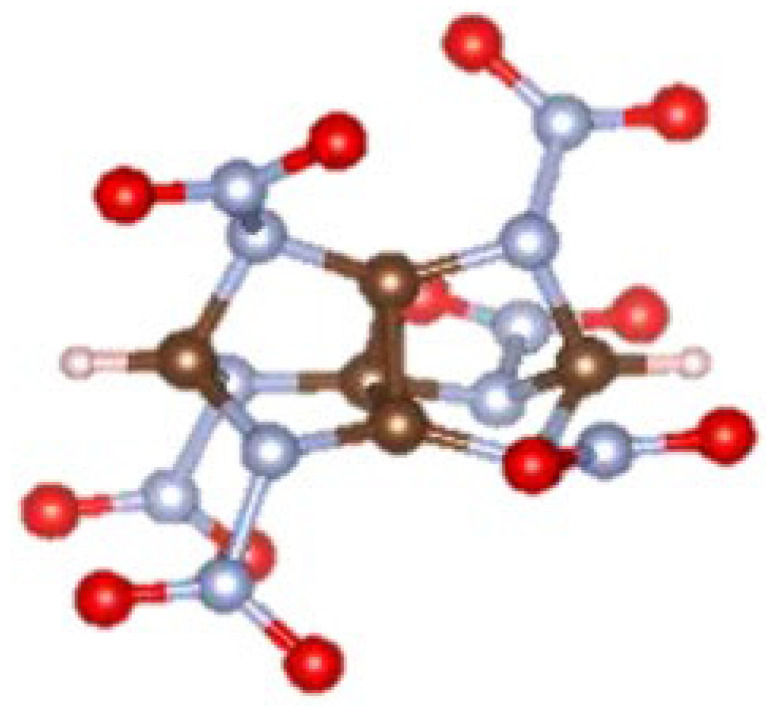
Molecular structure of molecule number164 [23]. Copyright 2021 Wiley-VCH GmbH.

As seen in Figure 12, 31 N-containing molecules, with high density, high detonation velocity and high detonation pressure, were screened. Among the 31 molecules, molecule of number 164 is new, which has not been reported before. The molecular structure of number 164 is shown in Figure 13.

As reflected in Figure 13, the molecule of number 164 has a cage-like structure similar to hexanitrohexaazaisowurtzitane (CL-20), of which the three detonation properties (density, detonation velocity, and detonation pressure) calculated by theoretical methods are all superior to those of CL-20 [23]. As a result of the establishment of suitable neural networks, the prediction errors have been effectively suppressed [23]. For example, the MAEs of crystal density, detonation velocity, and detonation pressure are 0.0259 g·cm^−3^, 0.3456 km·s^−1^, and 1.4933 GPa, respectively. The results [23] also showed that a training dataset volume of 300 is enough to achieve high-precision extended prediction based on the reasonable selection of sample structures.

**Figure 14:**
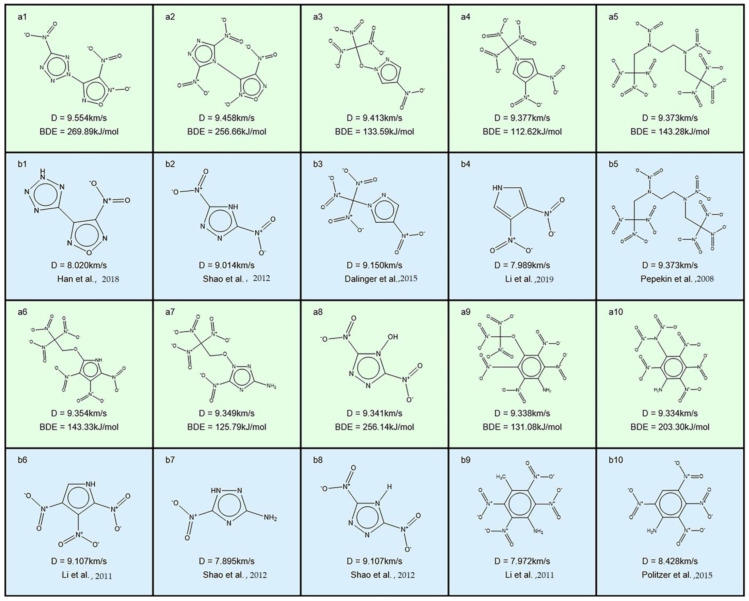
Structures of the top 10 molecules and similar compounds reported. The pale green and light blue backgrounds denote the molecules generated by Li et al. [79] and the similar molecules reported [112,113,114,115,116,117,118], respectively. D and BDE represent the detonation velocity and bond dissociation energy, respectively [79]. Copyright 2022 American Chemical Society.

Li et al. [79] developed RNNs to efficiently generate and screen novel EMs with a high detonation velocity and a low synthetic accessibility (SA) score. High-precision quantum mechanics calculations further confirmed that 35 new molecules present a higher detonation velocity and lower SA than RDX, along with good thermal stability. To further validate the advantage and the structural effectiveness of these promising candidates designed, Li et al. [79] selected the top 10 molecules in the detonation velocity order to correlate with related energetic works, as shown in Figure 14.

As shown in Figure 14, the 10 molecules generated exhibit some extent similarity to 10 energetic molecules previously reported, and the detonation velocities of the top 10 molecules fall in the range of 9334−9554 m·s^−1^, significantly superior to RDX (8927 m·s^−1^). In particular, the top three molecules present comparable or higher detonation velocities than complicatedly caged CL-20 (9455 m·s^−1^), along with a lower SA (SA of CL-20: 5.44). As is known, CL-20 has been the most powerful non-nuclear energetic compound in practice so far [79]. The results could provide helpful guidelines for applying the deep learning-based molecular design in R&D of EMs.

### 3.2. Composite EMs

In contrast to single-compound EMs, heterogeneous EMs have microstructures filled with voids, crack networks and other defects [68]. To some extent, the reverse design of composite EMs using ML methods may encounter more difficulties and challenges, compared to the R&D of single-compound EMs. Heterogeneities determine explosive performance behavior by triggering chemical reactions at hot spots or regions of localized heating [68]. In the discovery process of excellent heterogeneous EMs with tailored performance, it is necessary to create a linkage between micro-structural details and performance to guide the researchers. The heterogeneous compound made up of an inert polymer matrix and a high-loading fraction of an energetic organic crystalline powder was considered by Walters et al. [68]. By choosing the particle size distribution to optimize density, the researchers presented one part of an overall approach using the ML method to correlate particle size distribution with all of the key performance metrics [68].

In EMs formulations and designs, plasticizers and binders can be categorized into inert (non-energetic) and energetic [119]. Plasticizers are low molecular weight additives used to adjust the final polymer properties, and energetic plasticizers contribute to the overall formulation of energy by an increase in the enthalpy of the EMs system [119]. Sheibani et al. [119] used the molecular dynamics simulations and ML methods to determine the physicochemical and energetic properties of some novel azido-ester structures. Comparing experimental and theoretical results showed acceptable agreement between molecular dynamics simulations and ML methods. Finally, using the rheometry and differential scanning calorimetry analyses, the compatibility and efficiency of two novel azido-ester plasticizers on the rheological and thermal properties of glycidyl azide polymer (GAP) were investigated, and the two novel azido-ester plasticizers were also compared with some common energetic plasticizers. The results confirmed that these two novel azido-esters are appropriate plasticizers for GAP since they exhibited higher safety over comparable plasticizers [119].

A co-crystal is a single-phase crystalline material composed of two or more neutral molecules assembled by noncovalent forces in a specific proportion, which is neither a solvate nor a simple salt [8,120]. Zohari et al. [8] applied the QSPR method to examine the relationship between energetic co-crystal densities and their molecular structures. The research methodology provides a model that can relate the density of an energetic co-crystal to several molecular structural descriptors [8]. To integrate important prior knowledge into end-to-end learning on the molecular graph, a feasible GNN framework was also explored, and one novel energetic co-crystal predicted was successfully synthesized, showcasing the high potential of the GNN model in practice [72].

The energetic melt-castable material with promising properties was found through ML-assisted HTVS and experimental approaches [63]. In addition to high-throughput molecular generation, the ML-assisted HTVS system used five ML-based prediction models for predicting properties. Using this system, Song et al. [63] rapidly targeted 136 promising candidates of melt-castable material from a generated molecular space containing 3892 molecules. With extensive efforts on experimental synthesis, eight novel energetic melt-castable materials were obtained, and their measured properties were in good agreement with the predicted results [63].

**Figure 15:**
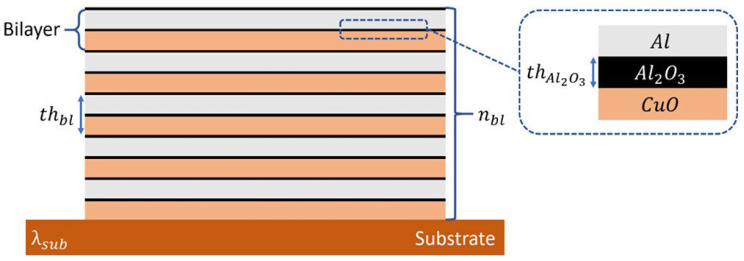
Schematic of the geometrical features of an Al/CuO nanolaminate deposited on a substrate [101]. Copyright 2022 American Chemical Society.

Nanothermites have attracted considerable interest in civil-military integration due to their unique properties. However, it is still challenging to predict quantitative structure-energetic performance relationships for nanothermites. To design novel nanothermites with optimal burning rates for a controllable energetic performance, Sami et al. [101] used ML methods to surrogate complex physical models. Nine supervised regression algorithms are compared and investigated for Al/CuO nanolaminates. The dataset contained a set of 2700 Al/CuO nanolaminate systems, which was used to construct an ML model for each regression algorithm [101]. Figure 15 shows the geometrical features of an Al/CuO nanolaminate deposited on a substrate.

Sami et al. [101] demonstrated that the multilayer perceptron algorithm could surrogate conventional physical-based models and reliably predict the Al/CuO nanolaminate microstructure-burn rate relationship. For example, by applying the multilayer perceptron algorithm, the burn rate of Al/CuO nanolaminate was estimated with less than 1% error (0.07 m·s^−1^), which is excellent considering that it typically varies from 8–20 m·s^−1^ for nanoengineered materials. In addition, the optimization of the Al/CuO nanolaminate structure for burn rate maximization occurred within a few milliseconds by using the ML method, versus several days by using the physical model, and months by experimentally optimizing it [101].

## 4. Challenges of Applying ML Methods

People have witnessed the emergence of the fourth paradigm of science represented by ML or artificial intelligence methods, probably partly owing to the big data generated by experiments and simulations in recent years [16,121,122]. It is now believable to predict material properties and optimize design materials with the help of ML methods. Although EMs can be predicted and screened using the ML method, some challenges still exist to overcome.

(1) In real-world scenarios, ML algorithms have been severely hindered by data acquisition challenges. Due to high costs, long cycle time, and safety concerns, collecting and/or accessing large amounts of data in the EMs area remains challenging. To some extent, applying data augmentation or the MF information-fusion approach using any arbitrary, randomly selected, molecular orientation during model training is an essential strategy. In addition, to improve the data quality, data cleaning is a standard procedure in the process of dataset preparation. However, problems such as inaccurate data in the literature or data pollution in the well-known database [123,124,125] should also be paid attention to.

(2) Chemists are still grappling with how to best feature molecules as inputs for ML models, whether by hand-crafted features or computer-learned representations. Regarding the traditional class of molecular representation, it is generally better to use models based on simpler molecular descriptors rather than those based on much more complex descriptors. It is reasonable that different molecular representations should be compared based on data and models to select the best one for specific problems. However, with the development of deep learning algorithms, the computer-learned representation may be the mainstream development trend in the future. To achieve accuracy, such deep learning methods require a large amount of training data, especially those with many tunable parameters. Thus, to realize a globally universal descriptor, it is essential to improve the existing descriptors and discover a universal descriptor for EMs.

(3) At present, most the research was focused on simple or traditional explosives, such as RDX, HNB, TATB, etc. Researchers have accumulated rich data and experience in feature extraction and other aspects for these simple or traditional compounds. It is urgent to develop and design high-energy and low-sensitivity compounds, including high-energy density materials, all nitrogen materials, and polymeric ammonia materials. Although the traditional ML or deep learning methods have shown promise for simple and traditional explosives, it is unclear to what extent they can be helpful in real-world advanced EMs development.

## 5. Summary and Outlook

Prediction and construction of advanced EMs based on the ML method have received more and more attention. In the properties’ prediction of EMs, the chemical composition of EMs is given as inputs, and the properties are predicted, which can be called the direct problem. In the inverse EMs design, the properties of EMs are the input, and the structure and composition are the output, which can be called the indirect problem. Among the direct and indirect problems, the most exciting problem is identifying promising chemical components and structures of EMs, which can be synthesized in the lab step-by-step. Theoretically, according to the ML model trained by a given dataset, the inverse design can be conducted to discover advanced EMs with regulated properties.

ML has a powerful ability indeed, but its establishment depends on sufficient training data, data augmentation strategy, etc. While existing databases contain a large amount of useful material data, more data are available in published papers that have yet to be entered into databases. Therefore, a more comprehensive and general material information standard should be established to make data sharing between databases and reduce obstacles to data acquisition. In terms of models or algorithms, the deep learning method is the mainstream development trend. In the most accepted format of the ML model, ML algorithms of different natures in a unified framework are needed, pivoting around the digital twin, to promote high-quality applications in the research field of Ems. Despite a substantial number of successful applications, the ML method is still largely in its infancy, and it is believed that it will play an increasingly important role in accelerating the development of advanced and novel EMs in the foreseeable future.

## Figures and Tables

**Table 1:** The common databases in the pieces of literature.

No.	Database Name	Sources
1	CCDC	[1,53,69,70]
2	GDB	[65,71]
3	CSD	[4,24,72,73,74]
4	PubChem	[72,73,75,76]

**Table 2:** A comparison of the prediction performance by the computer-learned representation and the traditional class feature extraction.

Model Category	Target EMs	Target Property	Main Method	Accuracy	F_1_ Score	Mean Absolute Error (MAE)	Root Mean Square Error (RMSE)	Determination Coefficient (R^2^)	Source
The classification model	Graphite-like layered crystal	Impact sensitivity	CNN	0.98	0.94	/	/	/	[1]
			LSTM	0.93	0.78	/	/	/	
			K-nearest neighbor (KNN)	0.95	0.33	/	/	/	
The regression model	HE	Density	Support vector regression (SVR)	/	/	/	0.085	0.683	[24]
			Random forests (RF)	/	/	/	0.053	0.878	
			Partial least-squares regression	/	/	/	0.048	0.9	
			Message passing neural network (MPNN)	/	/	/	0.044	0.914	
The regression model	Nitramines	Density	Group addition method	/	/	0.092	0.12	0.686	[70]
			Support vector machine (SVM)	/	/	0.097	0.122	0.796	
			RF	/	/	0.088	0.105	0.624	
			Quantitative structure−property relationship based on the DFT (DFT−QSPR)	/	/	0.041	0.057	0.941	
			GNN	/	/	0.04	0.047	0.944	
The regression model	CHNO-containi-ng energetic molecules	Detonation velocity	RNN	/	/	0.0968	0.1391	0.9445	[79]
			RNN model with inclusion of the pretrained knowledge (SRNN)	/	/	0.0801	0.1273	0.9572	
			RF	/	/	0.1812	0.2524	0.819	

**Table 3:** A list of important ML methods in the literature.

Method	Category	Target Property	Source
KRR	Regression	Density, detonation velocity, detonation pressure, decomposition temperature, heat of formation, heat of explosion, enthalpy of formation, burn rate	[1,13,73,101]
Least absolute shrinkage and selection operator	Regression	Density, molecular flatness, bond dissociation energy, heat of formation, heat of explosion, enthalpy of formation	[4,13,73]
Linear regression model	Regression	Heat of formation, heat of explosion, burn rate	[13,76,101]
Logistic regression	Regression	Heat of explosion	[76]
Multiple linear regression	Regression	Density, molecular flatness, bond dissociation energy, heat of formation	[4,8]
Gaussian process regression model (GPR)	Regression	Heat of formation, heat of explosion, burn rate	[13,101]
Artificial neural network (ANN)	Regression, classification	Detonation velocity, density, heat of explosion, bulk modulus, impact sensitivity	[64,74,102,103,104,105]
SVM	Regression, classification	Density, molecular flatness, bond dissociation energy, heat of formation, impact sensi-tivity, heat of explosion	[4,13,70,72]
SVR	Regression	Density, enthalpy of formation, heat of explosion, burn rate	[73,76,101]
CNN	Regression, classification	Graphite-like layered crystal structure, enthalpy of formation	[1,75]
RNN	Regression, classification	Detonation velocity	[79]
LSTM	Regression, classification	Density, detonation velocity, detonation pressure, decomposition temperature, enthalpy of formation	[1,75]
GNN	Regression, classification	Density, impact sensi-tivity, heat of explosion	[70,72]
Deep neural network (DNN)	Regression, classification	Impact sensi-tivity, heat of explosion	[72]
RF	Regression, classification	Density, molecular flatness, bond dissociation energy, heat of formation, enthalpy of formation, impact sensi-tivity, heat of explosion, burn rate	[4,70,72,73,76,101]
KNN	Regression, classification	Density, detonation velocity, detonation pressure, decomposition temperature, enthalpy of formation, burn rate	[1,73,101]
Multilayer perceptron (MLP)	Regression, classification	Burn rate	[101]
Decision tree	Regression, classification	Burn rate	[101]
High-dimensional neural network	Regression, classification	Binding energy, atomic force	[37]
Generative adversarial networks	Regression, classification	Porosity distribution	[52]
MPNN	Regression, classification	Density, impact sensi-tivity	[24,71]

## Data Availability

Not applicable.

## Abbreviations

EMs	energetic materials
R&D	research and development
ML	machine learning
DFT	density functional theory
HE	high explosives
CHNO	carbon, hydrogen, nitrogen, and oxygen
CSD	Cambridge Structural Database
CCDC	Cambridge Crystallographic Data Centre
OB	oxygen balance
SMILES	simplified molecular input line entry specification
RNN	recurrent neural network
MF	multi-fidelity
CNN	convolutional neural network
LSTM	long short-term memory
KRR	kernel ridge regression
GNN	graph neural network
KNN	K-nearest neighbor
SVR	Support vector regression
RF	Random forests
MPNN	Message passing neural network
SVM	Support vector machine
QSPR	Quantitative structure−property relationship
SRNN	RNN model with inclusion of the pretrained knowledge
ANN	Artificial Neural Network
MLP	Multilayer perceptron
PLSR	Partial least-squares regression
C−J	Chapman−Jouguet
H_50_	Values of the drop weight impact height
Q_NO2_	Mulliken net charges of the nitro group
MAE	Mean Absolute Error
RMSE	Root Mean Square Error
R^2^	determination coefficient
TNT	2,4,6-trinitrotoluene
*P*_e(TNT)_	TNT equivalent power index
HNB	Hexanitrobenzene
TATB	1,3,5-triamino-2,4,6-trinitrobenzene
HTVS	high-throughput virtual screening
ICM-104	7,8- dinitropyrazolo[1,5-a][1,3,5]triazine-2,4-diamine
LLM-105	2,6-diamino-3,5-dinitropyrazine-1-oxide
RDX	1,3,5-trinitro-1,3,5-triazinane
CL-20	Hexanitrohexaazaisowurtzitane
SA	synthetic accessibility
GAP	glycidyl azide polymer
